# First Assessment of Oral Iron Chelator HBED Increases Iron Excretion in Black Rhinoceros (*Diceros bicornis minor*)

**DOI:** 10.3390/ani15202987

**Published:** 2025-10-15

**Authors:** Kathleen E. Sullivan, Shana R. Lavin, Lori K. Warren, Natalie D. Mylniczenko, Shannon E. Livingston, Mitchell D. Knutson, Eduardo V. Valdes

**Affiliations:** 1 Disney’s Animals, Science and Environment, Disney’s Animal Kingdom^®^, Bay Lake, FL 34761, USA; shana.lavin@disney.com (S.R.L.);; 2 Food Science and Human Nutrition Department, University of Florida, Gainesville, FL 32611, USA; 3 Department of Animal Science, University of Florida, Gainesville, FL 32608, USA

**Keywords:** iron overload, chelation, iron excretion, diet, hemosiderosis, hemochromatosis, iron storage

## Abstract

Black rhinoceros (*Diceros bicornis*) face threats to survival due to poaching in the wild and an incomplete understanding of preventive health monitoring under human care. Black rhinos under human care develop iron overload disorder (IOD) which is associated with predisposition to other disease, compromised immune function, hemolytic crisis, and death. Management of IOD is challenging but has been mitigated in some cases with dietary intervention and phlebotomy and documented through serum biomarker evaluation. Chelation therapy to reduce iron is rare in rhinos partially because of limited product availability and route of administration. An iron-specific chelator HBED (N,N’-Di(2-hydroxybenzyl)ethylenediamine-N,N’-diacetic acid) was investigated for oral use in southern black rhinos (*n* = 3) after successful testing was performed with equids as a model. Using a cross-over design, we tested the efficacy of short-term HBED administration. HBED was dosed at 40 mg/kg body weight for 10 days and resulted in increased urinary excretion of iron but unaltered fecal iron excretion in rhinos compared to control trials. Two rhinos maintained blood chemistry and cell distribution considered normal for the species. The third rhino experienced a hemolytic event after stopping HBED administration in the same time frame and at the same dose as the two conspecifics but fully recovered. Careful monitoring and tapering the drug at the completion of treatment is warranted, especially if the rhino’s iron load is considered high. HBED’s potential to induce iron excretion safely, as well as prevent excessive dietary iron uptake, may significantly benefit the black rhino population under human care.

## Simple Summary

Black rhinoceros under human care develop iron overload disorder (IOD) which is associated with negative health consequences. Management of IOD involves minimizing iron into the body with diet and pulling iron out through either large-volume blood collection or a specialized iron binder known as a chelator. A cross-over study design in which three black rhinoceros were dosed for 10 days with the oral iron-specific chelator HBED resulted in increased urinary iron excretion compared to the control treatment. While all rhinos remained healthy during testing, one individual had a hemolytic event after stopping treatment, but she fully recovered. Tapering of the HBED dose before ending treatment would be recommended to avoid this complication. Overall, the chelator showed promise in aiding iron excretion in critically endangered black rhinos under human care.

## 1. Introduction

The black rhinoceros (*Diceros bicornis*) is a critically endangered species, primarily due to poaching, which has reduced the wild population by >90% since 1970 [1]. Approximately 240 black rhinos are managed by humans worldwide, with ~87 in North America [2]. Black rhinos under human care are predisposed to non-hemochromatosis iron overload disorder (IOD) with laboratory and histopathologic evidence of cellular injury, necrosis, and clinical signs similar to human iron overload disorders [3,4]. Consistently elevated iron biomarkers across time (including transferrin saturation and ferritin) indicate excessive accumulation of iron in black rhino [4]. This link is supported with data revealing all published necropsies in black rhino under managed care had moderate to severe iron deposition in multiple organs [5]. Under human care, black rhinos have been documented with diseases that have either been induced or exacerbated by IOD, leading to research into diagnostic, treatment, and prevention strategies [3,4,6,7].

While current theories on the physiology of iron overload in human-managed black rhino vary, the consensus is that feeding practices are a factor [4,8,9,10,11]. Plant species consumed by wild black rhinos have a wide range of iron concentrations but are lower than most black rhino diets offered under human care [12]. Thus, high iron concentrations in diets under human care, as well as the lack of natural tannins, phytates, and other compounds that can bind iron and reduce its absorption have been investigated as contributing factors for IOD in black rhino [4,13,14,15,16]. Whether excess iron is absorbed as a result of metabolic dysregulation or excessive available intake, the outcome is the same and results in iron exceeding physiologic requirements. Once absorbed, iron does not have a regular excretion route from the body; thus, it can only be removed through phlebotomy or chelation [17].

In order to advance the care of this critically endangered species under human care, the aim of this study was to investigate oral administration of the iron chelator N,N-bis(2-hydroxybenzyl)ethylenediamine-N,N–diacetic acid (hereafter, HBED) for its ability to induce iron excretion in black rhino. This chelator has been tested for toxicity and iron elimination efficacy in rats, non-human primates, dogs, and humans [18,19,20,21,22]. Additionally, we previously showed that short-term oral administration of HBED safely increased iron excretion in horses [23], the most appropriate digestive model for black rhino [24,25]. Thus, we expected short-term dietary supplementation with HBED to increase iron excretion in black rhinos.

## 2. Materials and Methods

### 2.1. Animals and Housing

Three southern black rhinos (*Diceros bicornis minor*) in the care of Disney’s Animal Kingdom^®^ (hereafter, DAK; Bay Lake, FL, USA) participated in the chelation and control trials. Two males, BR1 and BR2 (body weight (BW) of 1297 kg and 1143 kg), were born under human care. These animals were 14 and 16 years of age at the time of study and were at the facility for 14 and 13 years, respectively. They were considered clinically healthy and did not have IOD as serum iron biomarkers were consistently at levels comparable to those in wild rhinos and below critical cutoffs for mammalian species (<60% transferrin saturation, <400 ng/mL ferritin; [4,26]). These rhinos had undergone phlebotomies and were placed on a low iron diet, resulting in low circulating iron-related markers for over 2 years prior to the start of the study. The third rhino, a 17-year-old female (BW of 1337 kg), BR3, arrived at DAK after the study was initiated. This animal had been housed at multiple institutions previously. Based on iron biomarkers, this female rhino was considered iron loaded (91% transferrin saturation, 1615 ng/mL ferritin prior to study start). The study was conducted in the fall of 2014 with the two male rhinos and in the fall/winter of 2015 with the female rhino.

Rhino habitats included a cement-floored barn with adjustable-sized holding areas, as well as outdoor areas of exhibit space for each individual rhino with periods of time spent in both areas as part of typical husbandry practices. Animals had ad libitum access to water in all locations. Blood sampling was collected voluntarily from the medial saphenous vein. All housing and maintenance practices in this study remained unchanged from the normal daily routine of the black rhinos during the study periods.

The authors confirm that the ethical policies of the journal, as noted on the journal’s author guidelines page, have been adhered to and the appropriate ethical review committee approval has been received. This study was reviewed and approved with informed consent of rhino owners both by Disney’s Animal Care and Welfare Committee (DACWC IR1401 approved 23 January 2014), and the University of Florida Institutional Animal Care and Use Committee (UF IACUC #201308111 approved 10 November 2013).

### 2.2. Experimental Design

The effect of oral HBED administration on iron excretion, iron status, and bloodwork, (serum chemistries, mineral panel, vitamin E, and complete blood counts) was evaluated using a 2-period cross-over design comparable to the study design used previously in horses [23]. All three rhinos were randomly assigned to receive HBED or control for 10 days, followed by a minimum 10-day washout phase and reallocation to the opposite treatment for a second 10-day period. This design permitted all animals to serve as their own control. Blood samples were obtained on days 1 and 10 of each treatment period for evaluation of hematology and iron status. Representative samples of feces and urine were collected during the last 5 days of each treatment period (d 6 to 10) for measurement of nutrient concentration and excretion. During the last 3 days of each study period (d 8 to 10), all feed, orts, and voided feces were collected and weighed to calculate daily feed intake, fecal excretion, and nutrient digestibility. Behavioral and body weight monitoring occurred throughout the study.

### 2.3. Treatments and Basal Diet

Before the study began, the HBED (van Iperen International, Westmaas, The Netherlands) was tested at Atlantic Analytical Laboratory (Whitehouse, NJ, USA) for elemental analysis, to ensure it was the correct chemical compound, and at the Bergeron Laboratory at the University of Florida (Gainesville, FL, USA) for purity via nuclear magnetic resonance. The HBED was verified and found to have over 98% purity and considered safe for oral administration. Treatments consisted of either HBED mixed into a vehicle or the vehicle alone as the control. The HBED was administered at 40 mg/kg BW per day based on allometric scaling for metabolic BW of the dosage previously administered to rats/humans [21,27] and horses [23]. Body weight was measured using a livestock floor scale on d 0 of each period and HBED dosing adjusted accordingly for each period. For BR1 and BR2, the males, the required amount of HBED was weighed daily and mixed with a vehicle containing cold sweet potato puree (100 g), oatmeal (15 g), and maple syrup (10 g) to increase palatability and ensure complete consumption. This mixture was formed into a “treat ball” and split in half, with one half offered to the rhino at each of the twice daily feedings of a pelleted feed. A similar method of delivery was used for the control treatment, but the treat ball contained no HBED. Due to individual palatability preference, the female, BR3, would not consume the treat balls. Instead, half of the daily dose of HBED was mixed with 50 mL of double distilled water (>18 mega Ω purity) and delivered orally to the back of the mouth from a 60 mL syringe. This dose was immediately followed with a sweet potato, and then this rhino was given the twice-daily pelleted feed allotment. For the control treatment, this animal received the double distilled water via syringe as described.

Basal diets were comparable among rhinos, and diet and rotation schedule through the various enclosures remained unchanged from their normal routine throughout the study. Individual diet items were weighed daily for each animal, and consisted of a high fiber, low-iron pelleted feed (Mazuri Rhino Browser Rhino Cube 5Z1P; Mazuri PMI Nutrition International, St. Louis, MO, USA), each individual rhino’s preferred browse species (browse defined here as branches and leaves of trees cut for consumption), Timothy and Coastal bermudagrass hays, and a variety of produce (Table 1). Except for daily sweet potato, produce items were rotated through a weekly schedule with 2 to 3 items given each day.

The majority of feed items were offered twice daily to enclosures with access throughout the day. All pellets, and a majority of the browse, hay, and produce were offered on the cleaned cement floor of the indoor holding facility. Produce from the basal diet was used for training behaviors and as a reward after HBED dosing. Limited amounts of feed items were provided to rhinos while held in the outdoor exhibit area. This included a portion of the daily allotment of browse hung along a wall, and a portion of hay and produce placed on a large cement pad in the outdoor enclosure to minimize soil consumption. The iron concentration in the total diet for BR1, BR2, and BR3 was 142, 147, and 177 mg/kg DM, respectively, with differences mainly due to individual preferences for enrichment items (Table 2).

### 2.4. Sample Collection

Feed intake was measured daily for each 10 d treatment period. Samples of each feedstuff offered were collected daily and composited at the end of each treatment period for subsequent nutrient analysis. Additionally, any orts remaining were collected from enclosures and recorded daily and analyzed by day. Water samples were obtained daily from all waterers, as well as directly from hose faucets, and composited as one representative sample for each treatment period for analysis. Treat ball vehicles, both with and without HBED, were also analyzed for nutrient content.

Before the start of each study period (d 0) and at the end of each period (d 10), blood was collected via saphenous venipuncture and placed into tubes containing 3.2% buffered sodium citrate (for HBED concentration), serum separator clot activator gel (for serum blood chemistry and serum iron biomarkers), metal-free no-additive royal blue tubes (for trace minerals and vitamin E) or potassium EDTA (for complete blood counts and immediate processing). An extra blood collection was performed on d 1 of the HBED treatment period, 6 h after the first dose, to permit an analysis of HBED presence in the blood relative to time of dosing. All blood tubes were kept at room temperature until further processing. Tubes containing sodium citrate, no additive, and serum separator clot activator gel were centrifuged at 2000× *g* at 4 °C for 18 min within 1 h of collection, and plasma and serum were subsequently stored at −80 °C until analyzed.

Fecal and urine samples were collected the last 5 d of each 10-day treatment period. Voided feces were collected twice daily and composited prior to representative subsampling for subsequent nutrient analysis. Fecal samples were collected from indoor holding areas as quickly as possible after excretion, but if rhinos were in their outdoor enclosure, samples sometimes were not obtained for up to 6 h. Only fecal samples uncontaminated by the environment and internal to a fecal bolus were used for analysis of nutrient concentrations. Feces were collected during the last 3 days of each study period into tared wheelbarrows, weighed on a floor scale, and used to quantify total fecal output and subsequent nutrient digestibility. Collection of total feces proved challenging due to the rotation of animals among different enclosures, where at two specific instances animals defecated in areas with soil and performed treading, or used their feet to kick the feces and drag it into the soil. In these cases, collection of feces was still attempted to estimate total fecal excretion, but in the end these days were eliminated from mean digestibility calculations. Any contamination with soil was considered exclusion criteria. Additionally, due to the behavior and location of urination (spraying backwards in multiple locations), total collection and quantification of daily urine excretion was not possible. To evaluate urine composition, samples uncontaminated by debris or water were collected from the floor of their indoor holding barn using a 60 mL syringe.

### 2.5. Sample Analysis

All feed and fecal samples were analyzed by Dairy One Laboratories (Ithaca, NY, USA) for proximate composition, minerals, gross energy, acid detergent fiber, neutral detergent fiber, and acid insoluble ash using Official Methods of Analysis (AOAC) methodology [28]. Water samples were analyzed by Dairy One Laboratories as well, for mineral content, nitrates, and coliforms, using United States Environmental Protection Agency approved methodology [28].

Blood and serum samples were submitted to IDEXX Laboratories (Westbrook, ME, USA) and subsequently analyzed at the Diagnostic Center for Population and Animal Health (DCPAH; East Lansing, MI, USA) for analysis of complete blood cell counts (IDEXX), blood chemistry (IDEXX), trace minerals (DCPAH), and vitamin E (DCPAH). Serum total non-heme iron and total iron binding capacity (TIBC) analyses were performed by investigators using a colorimetric ferrozine procedure [29]. Transferrin saturation percentage was calculated by dividing serum non-heme iron by TIBC. Serum ferritin was analyzed at Kansas State Veterinary Diagnostic Laboratory (Manhattan, KS, USA) by sandwich Enzyme-Linked ImmunoSorbent Assay (ELISA) utilizing a polyclonal rabbit anti-horse ferritin antibody [30]. Urine iron was analyzed using the same colorimetric procedure used for the total non-heme iron in serum [29]. Measurement of HBED in sodium citrate plasma and urine was performed by Craft Technologies Laboratories, Inc. (currently Eurofins Craft Technologies, Wilson, NC, USA) using a procedure developed in the laboratory of Raymond Bergeron at the University of Florida (Bergeron personal communication, August, 2014). In brief, the procedure included plasma protein precipitation in acetonitrile, followed by HBED detection using reverse-phase HPLC on a Luna C18 (2) column (Phenomex, Inc., Emeryville, CA, USA), using HBED and iron-saturated HBED as standards.

### 2.6. Statistical Analysis

Using data from a study in 5 humans showing a 50 ± 17% increase in iron excretion with HBED supplementation [22], a power analysis showed that *n* = 3 rhinos were sufficient to detect a difference due to HBED treatment at an alpha level of 0.05 with >90% power (SYSTAT version 13; Systat Software Inc., Chicago, IL, USA). All data were analyzed using a mixed model ANOVA suitable for a cross-over design (proc mixed function; SAS version 9.4; SAS Institute, Cary, NC, USA). Complete blood counts and serum chemistry data were analyzed using time (d 1 vs. 10) as a repeated measure and covariance structures of heterogenous compound symmetry or compound symmetry based on Akaike Information Criteria (AIC) and finite-sample corrected Akaike Information Criteria (AICC) for best model fit. Treatment, period, time, and all interactions of the three were included in the model as fixed effects and rhino nested within day was used as random effect. The PDIFF option of the least significant difference test was used to separate means when the model was significant (α ≤ 0.05). Significant differences due to period were not included in the tables but were reported in the discussion when there were significant effects. Nutrient digestibility, intake and output data were also analyzed with a mixed model ANOVA with treatment and period as fixed effects and rhino as a random variable. Because iron load was greater in one of the rhinos before the study began, a separate ANOVA was performed to compare differences in the iron biomarker, ferritin, across treatments using historic iron load (high versus average) as a fixed effect. A Kenward Rogers adjustment for the degrees of freedom was used for all statistical analyses. Data are presented as least squared means with standard error except where otherwise noted. Differences were considered significant at *p* < 0.05 or as trends at 0.05 < *p* ≤ 0.10 and noted when physiologically relevant.

## 3. Results

All rhinos consumed 100% of the HBED or control vehicle offered. Nutrient composition of diets offered was similar among individuals (Table 2). On a dry matter (DM) basis, diets consisted primarily of hay, followed by pelleted diet, browse, and minor amounts of produce (Table 3). Limited quantities of orts remained from each individual during each study period and were predominantly bermudagrass hay. Drinking water was found to make no contribution to ingested iron and had no coliforms or *E. coli* contamination (Table 4) which could potentially compromise digestion.

During treatment, HBED supplementation had negligible effects on complete blood cell counts (Table 5) and blood chemistry variables (Table 6). Neutrophils as a percentage of white blood cells were higher when rhinos received HBED than control (*p* = 0.05); however, this difference was not apparent for neutrophil count (*p* = 0.38). Serum amylase also trended to be higher on the control treatment compared to HBED treatment (*p* = 0.09). Despite these changes, the numbers remained within reference ranges reported for black rhinos [31,32], as well as normal reference ranges for the horse (as per University of Florida Veterinary Diagnostic Laboratory).

Daily dry matter intake, fecal output, and digestibility varied among individuals, but were comparable when evaluated on a body weight basis (Table 7). Digestibility of dry matter, crude protein, and NDF on a body weight basis were not different between treatments (Table 7). Iron intake and excretion on a body weight basis also did not differ between treatments, and apparent iron digestibility was negative on both treatments (Table 8).

**Figure 1:**
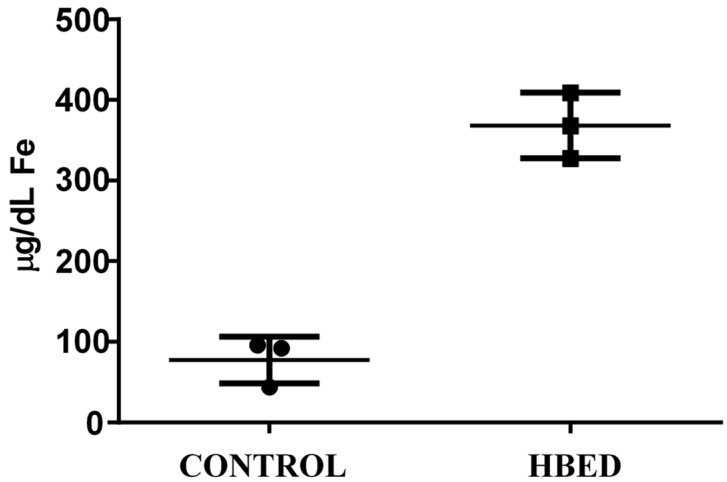
Urine iron concentration in *n* = 3 black rhino when supplemented with HBED or Control for 10 d. Values represent the mean ± SD urine iron for samples collected on d 5–10 of supplementation.

**Figure 2:**
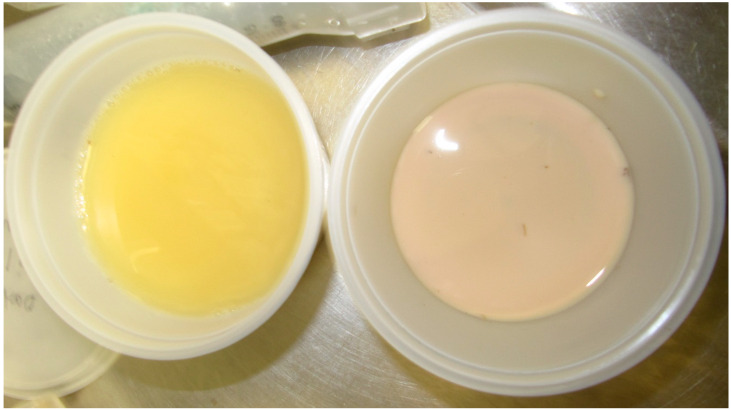
Visual comparison of rhinoceros urine when collected during control period (yellow in color on the left side of the photo) versus on HBED treatment (pink in color on the right side of the photo).

**Figure 3:**
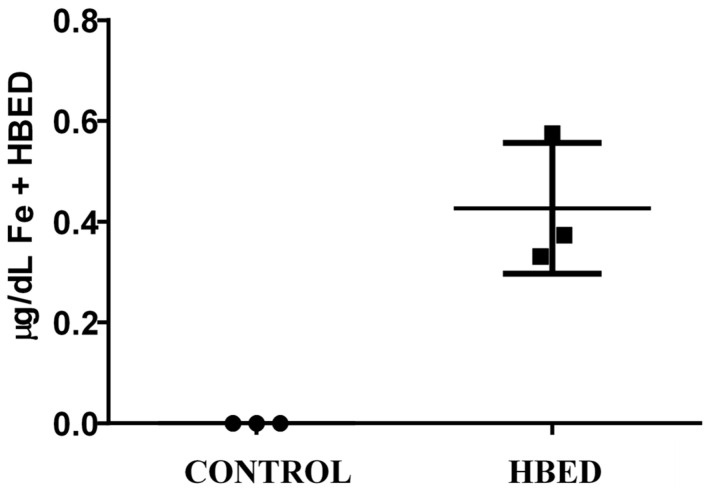
Concentration of HBED-bound iron in the urine of *n* = 3 black rhino when supplemented with HBED. Values represent the mean ± SD of urine samples obtained on d 6–10 of HBED supplementation.

**Figure 4:**
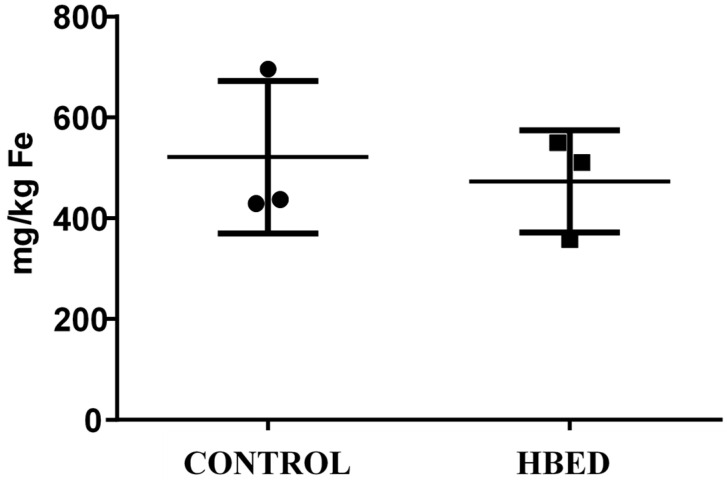
Fecal concentration of iron in *n* = 3 black rhino supplemented with HBED or Control for 10 d. Values represent the mean ± SD fecal iron for samples collected on d 5–10 of supplementation.

All rhinos excreted more iron in the urine when administered HBED versus control (*p* = 0.006; Figure 1), while fecal concentration of iron was the same under each condition (Table 8). Urine was also visually observed to be pink in color on HBED treatment, but yellow on control (Figure 2). Although HBED was not detected in plasma, HBED was found bound to iron in urine during HBED supplementation (Figure 3). Iron concentration and total daily iron excretion in feces varied among individuals when rhinos received HBED but did not differ from control treatments (*p* = 0.75; Figure 4).

Concentration of serum iron, TIBC, and transferrin saturation were not different between control and HBED treatments (Table 9). Higher TIBC was seen on d 10 than d 1 on both treatments (*p* = 0.01). Due to the higher iron load in the female rhino, a separate comparison of plasma ferritin between the female, BR3, and the male rhinos, BR1 and BR2, was performed across treatments. BR3 trended with higher ferritin than BR1 and BR2 (*p* = 0.09). As a result, a comparison of control versus HBED was evaluated in BR1 and BR2 across both time points, where it did not differ between treatments (Table 9). Although statistical analysis of treatment in BR3 was not feasible, plasma ferritin on d 10 of HBED treatment was 8-fold higher than d 1 of this treatment, and 11-fold higher than d 10 of the control treatment (Table 9).

Case note: Post HBED hemolysis in BR3.

At the end of HBED treatment, the two males, BR1 and BR2, appeared unchanged and had normal hematology. The female, BR3, however, had red transparent serum on the day after ending HBED treatment. This initial post-treatment sample did not correspond with hemolysis (hematocrit 37%) and the color was attributed to the presence of HBED, as the serum was normal in color with samples prior to HBED dosing and all animals had red-tinted urine and feces throughout the HBED dosing period. Four days after the cessation of HBED treatment, BR3 began showing signs of lethargy and partial anorexia and a repeated sample showed a moderate drop in hematocrit to 27% but otherwise normal bloodwork. This rhino also showed a drop in serum phosphorus from 3.2 mg/dL to 1.9 mg/dL one day after cessation of treatment. The phosphorus was already considered moderate to low, ranging from 2.4 mg/dL to 3.8 mg/dL over the course of the previous year. Over subsequent days, BR3′s serum continued to have a red color (at this time attributed to both HBED and hemolysis), which confounded some blood parameters due to assay interference; urine color also remained a red to pink color, as it had throughout the HBED treatment. Hematocrit continued to decrease steadily from day 4 through day 17 to 16% where it plateaued and then slowly started to increase. This increase occurred concurrently with oral supplementation of monosodium phosphate beginning on day 7 at 20 g per day (a total of 5 g of phosphorus) with 6 g of complexed phosphorus pills given as a one-time ‘boost’ also on day 7 post cessation of HBED treatment. Serum phosphorus changed from a low level of 1.9 mg/dL to a maximum level of 6.9 mg/dL twenty days after ending HBED treatment. Vitamin E in the form of Emcelle tocopherol (Stuart Products Inc., Bedford, TX, USA) was also added to the diet of BR3 at the same time oral phosphate treatment began, as we found vitamin E values of 0.13 µg/mL in this animal, compared to a clinically normal black rhino value of 1.0 µg/mL [32]. Bloodwork of BR3 during this time was monitored daily until HCT was stabilized and returned to normal at 7 weeks post cessation of HBED treatment. This female rhino fully recovered to a normal health status after this event.

## 4. Discussion

To advance the welfare and health of black rhinos, iron overload disorder needs to be properly managed, ideally by prevention or alternatively with treatment. Feeding practices for rhinos under human care are inevitably contributing to iron loading in this species (4). Methods to decrease iron ingestion and bioavailability remain the best options to lower risks of IOD in this species. Synthetic chelation therapy targeted specifically for iron has been used successfully in treatment of human iron overload diseases and has not been extensively investigated in rhinos, with the exception of a single successful treatment case of intramuscular injection of deferoxamine in a black rhinoceros in Japan [33]. The potential of HBED to induce iron excretion safely, as well as prevent excessive dietary iron uptake in this iron-overloaded species, would benefit management of the population under human care. Our objective was to investigate the effects of oral administration of the iron chelator HBED in black rhino, and we found that this compound indeed induces urinary iron excretion.

Success of treatment was judged by greater excretion of iron compared to the control. Urine iron concentration was increased in all rhinos on HBED treatment compared to control, though fecal iron concentration and output was not different between treatment and control. The increase in iron excretion in black rhinos shows great potential for management of IOD. In contrast to all other minerals, iron cannot be actively excreted from the body (as reviewed in detail in [34]). Recent studies have documented the possibility of minute iron excretion via the bile of mice, potentially upregulated in inflammatory conditions; however, this has not been translated into appreciable and active excretion that would combat IOD [35,36]. In humans, approximately 1–2 mg of iron, about 5% of total iron needs, is lost daily through desquamation of enterocytes and subsequent excretion in the feces, as well as through menstruation in females [34,37]. Whole-body iron homeostasis is maintained by the hormone hepcidin, which controls iron recycling and allows regulation through absorption of 1–2 mg of iron from the diet in the small intestine [17].

Although daily urine excretion could not be quantified, it was roughly estimated by using known urinary output from horses, the closest taxonomic relative. Horses supplemented with HBED in our previous study had a 0.7% increase in iron excretion via the urine compared to total iron intake, producing amounts of loss on par with human chelation targets [23]. Using a published estimate of urine output of 1.6 kg/100 kg BW in horses [38], the rhinos excreted an average of 77.0 mg/day of iron in the urine when receiving HBED versus 14.8 mg/day when on the control treatment, indicating HBED is increasing the relative amount of iron in the urine by at least 2.1% considering total iron intake. Chelation goals for humans on IV deferoxamine are an additional 20–50 mg of iron excreted through combined urine and feces daily (600–1500 mg per month; [39]). These may be conservative goals compared to the size of the black rhino, but indicate effective action to reduce iron load in the body.

Despite greater iron loss through urine, serum iron biomarkers did not change in response to short-term HBED treatment in the male rhinos, which was similar to our previous findings in horses [23], as these rhinos were not overloaded with iron. A longer period and/or higher concentration of daily dosing would potentially continue to promote iron excretion and could generate improvements in serum biomarkers of iron status in rhinos with IOD. A series of cases in dolphins has demonstrated that longer-term HBED treatment at a maximum dose of 80 mg/kg BW (6 to 18 months) created profound changes in ferritin and transferrin saturation, reducing values to within normal range for mammals [40]. In another case series, metallic starlings with iron storage disease were dosed for 4–6 months with oral HBED up to 100 mg/kg BW without changes in iron biomarkers including liver biopsy, although behavioral improvements were noted (Pers. comm. Sullivan and Mylniczenko). Also, a single case study dosed a Malayan flying fox with oral HBED for 4 months, documented urinary excretion of iron bound to HBED, and decreased serum transferrin saturation below 60% (Pers. comm. Sullivan and Mylniczenko).

The lack of fecal iron excretion finding is consistent with our previous study in horses, the latter of which enabled a more complete quantification of daily fecal and urine excretion through use of a collection harness [23]. Oral dosing of HBED with food induced iron excretion in humans mainly via feces, but also via urine in rats [21,22]. Iron excretion via urine implies HBED was absorbed to some degree in the gastrointestinal tract (GIT). HBED forms a 1:1 complex with ferric iron with high selectivity and affinity whether in the GIT or after absorption within the body [41,42,43]. If HBED was absorbed, first-pass metabolism would bring it through the liver, allow potential binding, and then go to excretion. However, HBED was not detected in the plasma of rhinos in the current study, nor in horses tested previously [23]. HBED was found both bound and not bound to iron in the urine, supporting that at least a fraction was absorbed in the GIT (Figure 3).

The absence of HBED in plasma of horses and rhinos may have several explanations. First, it is possible that the serial blood sampling performed after dosing in horses did not capture the window of absorption. However, this is unlikely as circulating HBED was not detected in any of the blood samples obtained from horses or rhinos spanning 1 to 12 h post dosing [23]. Sampling frequency was based on human and non-human primate models of HBED supplementation, as well as expected foregut digesta passage kinetics in horses [44]. Second, it is possible the plasma contained an HBED metabolite(s), which was not detected with the current analysis. First-pass metabolism through the liver certainly could have resulted in the formation of an HBED derivative excreted by the kidneys.

Plasma may also have had levels of HBED below the detectable limit of the assay. Considering the blood volume of a horse or rhino, it would be easier to detect HBED in concentrated urine. Rats treated with radioactive-labeled iron and intraperitoneal-injected HBED found fecal iron originated from liver storage pools, and iron excreted in the urine originated from processing of transfused red blood cells by the reticuloendothelial system (RES) cycle [22]. The origin or pathway of excreted iron with oral administration of HBED has not been studied. However, if less than 5% of orally ingested iron is absorbed, one could expect very little fecal iron to originate from the body [45]. Oral administration of deferoxamine, a synthetic hexadentate ligand like HBED, has been shown to enterally bind unabsorbed iron [20]. Therefore, HBED should bind iron in the GIT, with the majority of both dietary iron and HBED excreted in the feces. We were not able to analyze HBED in fecal excreta. While there is a lack of knowledge on rhino-specific absorption and regulation of iron homeostasis, the primary route of iron excretion would most likely be the feces, as reflected in our fecal excretion data. This is not surprising considering primary regulation of iron occurs at the absorption level, and dietary iron far exceeds biological need [34].

Some practical challenges of animal housing resulted in the inability to collect a full set of samples for determining nutrient digestibility. Rhinos treading the feces into the dirt in the outdoor enclosure disrupted quantification of daily fecal excretion and resulted in the removal of two samples from the dataset. Our observation of high fecal iron excretion that surpassed iron intake indicated potential unknown sources of iron intake or fecal contamination which has been reported previously [24]. Rhinos were not observed to consume soil, though they were not under constant observation so this possibility also cannot be eliminated. A portion of the browse offered on exhibit was often knocked to the ground before being consumed, potentially leading to contamination with soil as an extra source of elemental iron. This may help explain how an average of 2.4 mg iron/kg BW was consumed from the recorded diet and an average of 7.9 mg iron/kg BW (DM basis) was excreted in the feces, creating high negative digestibility estimates for both treatments.

Short-term treatment with HBED did not alter blood chemistry, serum minerals, or complete blood cell counts, indicating its relative safety (Table 6). Especially of note was the lack of change in non-iron microminerals, such as zinc and copper, which have binding potential with many chelators, especially natural forms like tannins [15]. Both male rhinos started this study with iron within range of wild counterparts, indicating a low iron load, and remained healthy throughout [4,26]. The lack of significant change in serum non-heme iron, transferrin saturation, and ferritin in the plasma in the males indicates that this short-term study, while increasing iron excretion successfully, did not compromise circulating iron in healthy animals. This finding is consistent with previous results in horses supplemented with HBED for 8 days [23]. The female rhino, however, was iron overloaded at the start of the study. During treatment, blood work remained consistent, but after cessation of treatment, this rhino experienced hemolysis that resolved with supportive treatment.

Case note discussion: Post HBED hemolysis in BR3

Clinically healthy serum iron biomarkers in black rhinos under human care include iron saturation under 60% and ferritin levels close to wild counterparts (290 ± 18 ng/mL; [4,26]). Through previous dietary and phlebotomy interventions, the male black rhinos entered the current study with iron profiles that were not considered excessive [4,16]. The male rhinos also had normal serum phosphorus (3.1 and 3.2 mg/dL), but relatively low serum vitamin E (0.47 and 0.26 µg/mL). In contrast, the female black rhino (BR3) began this study with relatively low serum phosphorus for a black rhino (2.3 mg/dL; “normal” range 2.5–3.9; [31]), a lower concentration of serum vitamin E (0.13 µg/mL), and higher iron load than that observed in wild black rhinos (1615 ng/mL ferritin; [26]). This female maintained normal behaviors, appetite, and treatment compliance throughout control and HBED administration. It should be noted that although this rhino was offered a diet with almost twice as much iron content, she was an extremely selective eater, resulting in daily iron intake similar to the male rhinos (Table 7). As described in the results, four days after concluding the study and HBED treatment, this rhino exhibited signs of hemolysis that were resolved with treatment. Elevated serum ferritin (14,158 ng/mL) observed on the final day of HBED treatment had returned to its control treatment level (1733 ng/mL) by the following month. This hemolytic crisis appeared to initiate ferritin synthesis in response to inflammatory processes in its function as an acute phase protein, as well as its role in containing excessive iron in circulation [46]. One month after concluding HBED treatment in the female rhino, the hemolysis was resolved, an average of 43 ± 7% transferrin saturation was maintained, and typical activity for the next 3 months resumed with no further intervention warranted.

The morbidity event observed in BR3 following cessation of oral chelation treatment appeared to be related to multiple factors. The development of severe anemia and leukocytosis raised concerns about an infectious process being involved but was not confirmed. It is uncertain whether preexisting conditions, including high iron load and low antioxidant and phosphorus status, were influential in an immune response resulting in acute hemolysis. Black rhinos have unique physiological differences that could have further confounded her diagnosis. For example, black rhinos have documented low activities of catalase and glutathione S-transferase enzymes, as well as adenosine triphosphate (ATP) levels at 2–5% of the concentrations found in most mammalian erythrocytes [47,48]. Normal physiology of rhino red blood cells (RBCs) results in diminished antioxidant capacity, with symptoms similar to the genetic mutation in glucose-6-phosphate dehydrogenase (G-6-PD) deficiency in humans [3]. Black rhinos appear to respond to similar preventive and therapeutic strategies used for humans with G-6-PD deficiency [49]. This includes phosphate supplementation to stimulate ATP production in black rhino, which in part has been credited with a huge decrease in hemolytic episodes in black rhinos under human care [3] and may have influenced resolution in this case. In the 1980′s, hemolytic syndrome in black rhinos under human care had a 75% mortality rate, while at present there has been one case in the last ~20 years [3,50,51]. The need for antioxidant and phosphate supplementation to maintain physiological homeostasis in black rhinos under human care factors into dietary management of this species [4]. This need likely played a role in BR3′s vulnerability to hemolysis; due to selective eating habits and recent arrival to the DAK collection, BR3 had not yet begun a supplemental vitamin E regimen, nor was she supplemented with monosodium phosphate before this event.

Another possible explanation leading to the female rhino’s morbidity was the abrupt cessation of HBED at the conclusion of the study, rather than a tapered dose (tapering is not a standard practice in human medicine). Effective chelation therapy may have left reactive and unbound iron mobilized from either the liver or RES, consequently triggering a reactive oxidant cascade [52,53]. In this iron-overloaded rhino, it is possible that turnover and excretion of excess iron from labile iron pools was extremely effective, mobilizing and excreting iron through the kidneys. After 10 days of regular dosing, the sudden absence of HBED may have led to an imbalance between effective chelation and iron excretion, producing an excess of released labile iron. This cascade did not occur in the males, but iron load was notably low and thereby potentially less reactive in these animals. However, if the HBED dose had been tapered to zero in BR3, it would potentially allow downregulation of iron mobilization and release with the HBED chelation. HBED has been shown as protective rather than stimulatory of several reactive oxidant species so its continued presence might mitigate reactive chemistries [54,55]. Based on the evidence of this case, we would recommend tapering to zero in future studies utilizing oral HBED.

A plausible explanation for the events observed in BR3 is that the released iron no longer had a binding agent after the abrupt cessation of HBED and was left as free labile iron. This iron would serve as a free radical, making it reactive in redox (Fenton) chemistry with the potential to catalyze the formation of superoxide and hydroxyl radicals [56,57,58]. These, in turn, can create chain reactions of oxidation, destroying cells, especially red blood cells in individuals with iron overload-related disease states [59]. The acute phase protein ferritin seemingly responded to this inflammatory oxidant process and hemolysis by elevating 10-fold in BR3. Ferritin, as the primary containment and storage protein utilized by macrophages in the RES, would be upregulated in times of sudden changes in circulating iron from sudden hemolysis. It should be noted that the other causes of elevations in ferritin involve chronic inflammation stemming from increased cytokines, chronic liver damage causing release from destroyed hepatocytes, and malignancies from destroyed tumors [60].

## 5. Limitations

While all animals appeared healthy throughout this study, the morbidity event observed in the female rhino (shortly after concluding the study) cannot be extricated from possible effects of HBED administration. The male rhinos did not have the same experience following HBED treatment. This may be because of sex differences, but more likely the males’ lower iron load led to differences in chelation response in the body. Further study, including both sexes, would be beneficial as well as standardized tapering, especially if there is evidence of iron overload. While we established 90% prior power and strengthened our mixed-model analysis using a cross-over design, the limited number of rhinos tested should be considered when applying HBED treatment to the rhino population at large. There were noted challenges to collection of excreta including the need for urine aliquots from a clean floor; therefore, horse output estimates were used in urine calculations. Potential soil intake and browse soil contamination may connect to negative iron digestibility results.

## 6. Conclusions

While HBED appeared to increase iron sequestration and excretion via the urine, complications in the female rhino raised concern about the treatment protocol in iron-overloaded individuals with regard to abrupt termination of the HBED. If the BR3 had been treated until the iron load was normalized, the cessation may not have resulted in this course, as it did not in the normal BR1 and BR2. We recommend tapering off the dose for any animal that began treatment with an iron load. The speed of tapering (whether over one week or more) may depend on the individual’s iron and overall health status.

If future work used a protocol similar to the one in the current study, we would recommend beginning at 40 mg/kg BW HBED with close monitoring of ferritin, transferrin saturation, and CBC (as per Methods). All rhinos should be assessed and ideally corrected for optimal phosphorus and vitamin E status before and during chelation. In iron-loaded animals, a gradual taper rather than abrupt cessation seems prudent. We would recommend planning future studies to take advantage of a period of time when rhinos would be held indoors, such as cold weather, to allow for less potential soil contamination. Ideally, mixed sexes and a larger sample size across multiple institutions utilizing the same withdrawal protocol is recommended in future studies.

Another consideration for investigation would be to administer a long-term low-level dosage, although efficacy would depend on the individual’s iron load, as chelation is generally dose dependent. There is demonstrated efficacy of this chelation treatment that could potentially prevent or manage iron overload in black rhino under human care; however, careful monitoring of heavily iron-loaded individuals is recommended. In treatment using HBED in bottlenose dolphins [40], long-term HBED dosing was successful with very rigid monitoring parameters as the animals were also heavily iron loaded, and a slow taper was employed once iron levels stabilized. While iron overload disorder threatens the health and survival of black rhinos managed under human care, HBED, and chelation in general, may be a potential treatment strategy.

## Figures and Tables

**Table 1:** Composition of basal diets (g/d as fed) offered to individual black rhinos.

	Individual Black Rhino		
Feed Item	BR1	BR2	BR3
Browse			
Ear Leaf Acacia	0.0	0.0	15,800.0
Acacia Longifolia	0.0	0.0	11,450.0
Elaeagnus—Silverberry	9400.0	9700.0	0.0
Hay			
Bermudagrass	9500.0	9600.0	8200.0
Timothy	6200.0	8600.0	11,900.0
Pelleted feed			
Browser Rhino Cube 5Z1P	6200.0	6200.0	10,400.0
Treat Ball Vehicle	200.0	200.0	0.0
Daily Enrichment			
Sweet potato	758.0	458.0	1078.0
Romaine	1500.0	896.0	1325.0
Training Items ^†^			
Cucumber	0.0	360.0	0.0
Green bean	330.0	0.0	0.0
Yellow squash	340.0	0.0	0.0
Celery	690.0	0.0	0.0
Bok choy	1500.0	304.0	1523.0
Apple	186.0	186.0	372.0
Pinecone	33.3	66.6	33.3
Carrot	0.0	360.0	200.0
Kale	0.0	0.0	1850.0
Banana with peel	0.0	0.0	210.0
Green plantain with peel	0.0	0.0	340.0
Total offered per day without training items	33,758	35,654	60,153

^†^ Two to three different training items were offered daily on a rotating schedule congruent with normal husbandry practices for each individual black rhino.

**Table 2:** Mean nutrient composition (DM basis) of the total diet fed daily to individual black rhino.

		**Individual Black Rhino**		
**Nutrient**	**Unit**	**BR1**	**BR2**	**BR3**
Dry Matter	%	66.03	73.25	77.61
Gross Energy	kcal/g	4.59	4.59	4.46
Crude Fat	%	3.04	3.01	2.95
Crude Protein	%	12.01	11.86	12.09
Starch	%	2.54	2.41	3.37
Lignin	%	6.80	6.66	4.44
Acid Detergent Fiber	%	36.97	37.28	34.45
Neutral Detergent Fiber	%	61.20	61.30	56.32
Ethanol-Soluble Carbohydrates	%	7.13	6.99	6.69
Water-Soluble Carbohydrates	%	8.79	9.24	10.76
Ash	%	6.42	6.40	7.38
Calcium	%	0.84	0.83	0.89
Cobalt	mg/kg	0.40	0.39	0.40
Copper	mg/kg	7.31	7.27	7.76
Iron	mg/kg	147.09	141.61	176.51
Magnesium	%	0.22	0.22	0.24
Manganese	mg/kg	124.52	118.32	61.01
Molybdenum	mg/kg	1.04	1.07	1.15
Phosphorus	%	0.23	0.22	0.25
Potassium	%	1.35	1.35	1.52
Selenium	mg/kg	0.25	0.24	0.33
Sodium	%	0.23	0.21	0.31
Sulfur	%	0.28	0.27	0.30
Zinc	mg/kg	61.10	58.00	74.68
Vitamin A	IU A/g	9.37	8.66	7.85
Ascorbic Acid	g/kg	13.10	12.43	10.33
Vitamin E	mg/kg	294.91	272.89	88.84

**Table 3:** Representative comparison of diet composition on a dry matter and as-fed basis for one black rhino (BR2).

				Hay		Browse
	Units	Produce	Pellet	Timothy	Bermudagrass	*Elaeagnus* sp.
As Fed	% ^†^	6	17	24	26	27
Dry Matter	% ^†^	2	20	29	33	16

^†^ Percent of total daily ration.

**Table 4:** Analysis of water samples collected from black rhino holding facilities.

Analysis	Units	Average ^†^	Standard Deviation
pH		7.97	0.09
Total Coliform	/100 mL	0.00	0.00
*E. coli*		Negative	Negative
Nitrates	mg/L	3.33	3.33
Nitrates—Nitrogen	mg/L	0.67	0.67
Sulfates	mg/L	2.67	2.67
Sulfates—Sulfur	mg/L	1.00	1.00
Chlorides	mg/L	14.67	1.20
Hardness (CaCO_3_)	mg/L	148.00	30.73
Total Dissolved Solids	mg/L	237.00	50.33
Calcium	mg/L	41.79	8.17
Phosphorus	mg/L	0.04	0.02
Magnesium	mg/L	10.68	2.54
Potassium	mg/L	8.95	5.49
Sodium	mg/L	8.77	0.70
Iron	mg/L	0.00	0.00
Zinc	mg/L	0.04	0.04
Copper	mg/L	0.00	0.00
Manganese	mg/L	0.00	0.00
Molybdenum	mg/L	0.01	0.00

^†^ Samples were collected from various water sources and composited in each study period (*n* = 4).

**Table 5:** Mean blood cell distribution in *n* = 3 black rhino supplemented with HBED or control. Values represent the least squared mean of samples obtained on d 10 of supplementation.

	Unit	Reference Range ^†^	Control	HBED	SEM	*p*-Value
White Blood Cells	K/μL	5.6–11.6	5.5	6.6	1.4	0.63
Red Blood Cells	M/μL	6.6–11.0	4.7	4.6	0.3	0.26
Hemoglobin	g/dL	11.0–16.0	14.2	13.7	0.6	0.36
Hematocrit	%	30.0–44.0	38.6	38.4	1.5	0.92
Mean Corpuscular Volume	fL	38.0–51.0	83.5	85.5	5.5	0.79
Mean Corpuscular Hemoglobin	pg	13.0–19.0	30.5	30.3	1.1	0.71
Mean Corpuscular Hemoglobin Concentration	g/dL	35.0–39.0	36.7	35.6	1.1	0.55
Neutrophil	%	-	66.3	77.8	3.6	0.05
Lymphocyte	%	-	22.5	16.8	3.2	0.14
Monocyte	%	-	7.3	3.3	0.8	0.13
Eosinophil	%	-	3.9	2.1	0.5	0.15
Basophil	%	-	0	0	0	-
Neutrophil	K/μL	2.6–6.7	3.7	5.1	1.0	0.38
Lymphocyte	K/μL	1.1–5.7	1.3	1.1	0.4	0.84
Monocyte	K/μL	0–0.7	0.4	0.2	0.1	0.18
Eosinophil	K/μL	0–0.6	0.2	0.2	0.1	0.41
Basophil	K/μL	0–0.2	0	0	0	-

^†^ Reference range provided is for equine from the University of Florida Veterinary Diagnostic Laboratory, as no normal range exists for black rhinos.

**Table 6:** Blood chemistry and serum trace minerals in *n* = 3 black rhino supplemented with HBED or control. Values represent the least squared mean of samples obtained on d 1 and d 10 of supplementation.

			Treatment			*p*-Value		
Variable ^¶^	Units	Reference Range ^†^	Control	HBED	SEM	Trt	Time	Time × Trt
ALP	U/L	69–228	53.9	46.0	5.4	0.38	0.37	0.02
ALT	U/L	-	6.2	4.1	3.8	0.72	0.64	0.64
AST	U/L	148–322	45.5	36.7	18.8	0.76	0.19	0.77
Creatine Kinase	U/L	-	165.3	150.6	78.2	0.9	0.79	0.21
GGT	U/L	17–50	19.0	28.7	7	0.4	0.30	0.39
Amylase	U/L	-	1.8	1.0	0.2	0.09	0.42	0.46
Lipase	U/L	-	7.0	8.1	1.4	0.6	0.71	0.39
Albumin	g/dL	2.7–4.5	2.3	2.4	0.1	0.22	0.44	0.83
Total Protein	g/dL	6.1–8.4	7.2	7.3	0.2	0.74	0.44	0.79
Globulin	g/dL	2.4–4.9	4.9	5.0	0.2	0.85	0.64	0.65
Total Bilirubin	mg/dL	0.3–1.9	0.1	0.2	0.1	0.66	0.41	0.54
BUN	mg/dL	9.0–22.0	11.2	10.6	0.3	0.22	0.21	0.61
Creatinine	mg/dL	1.1–2.0	0.8	0.8	0.01	0.22	0.44	0.97
Cholesterol	mg/dL	-	68.5	58.8	9.1	0.51	0.90	0.73
Glucose ^‡^	mg/dL	62–128	60.9	64.0	3.0	0.51	0.01	0.39
Albumin/Globulin		0.6–1.4	0.44	0.48	0.02	0.46	0.37	0.50
Triglyceride	mg/dL	4.0–44.0	5.9	9.5	6.8	0.73	0.26	0.27
Osmolality	mOsm/kg	-	281	283.5	2.2	0.47	0.62	0.32
Phosphorus	mg/dL	2.3–4.7	2.8	3.0	0.3	0.62	0.09	0.65
Calcium	mg/dL	10.7–13.3	11.8	11.8	0.1	0.46	0.50	0.86
Magnesium	mg/dL	1.2–1.9	2.6	2.5	0.1	0.7	0.33	0.73
Sodium	mmol/L	136–144	130.8	131.3	0.9	0.74	0.70	0.26
Potassium	mmol/L	2.2–5.3	4.3	4.6	0.3	0.5	0.17	0.85
Chloride	mmol/L	96–105	95.5	97.2	1.3	0.41	0.42	0.31
TCO_2_	mmol/L	22–30	20.2	17.3	1.8	0.37	0.33	0.50
Vitamin E	mmol/L	-	0.5	0.3	0.1	0.41	0.39	0.18
Cobalt	μg/mL	-	0.7	0.8	0.3	0.83	0.21	0.06
Copper	ng/mL	-	1.2	1.3	0.03	0.51	0.46	0.46
Iron	μg/dL	-	238.1	242.9	23.3	0.89	0.29	0.90
Manganese	ng/mL	-	24.3	0.0	15.2	0.32	0.43	0.43
Molybdenum ^§^	ng/mL	-	11.9	11.1	1.4	0.7	0.02	0.82
Selenium	ng/mL	-	112	114.8	2.6	0.52	0.15	0.61
Zinc	ng/mL	-	0.9	0.9	0.04	0.93	0.73	0.65

^†^ Reference range provided is for equine from the University of Florida Veterinary Diagnostic Laboratory, as no normal range exists for black rhinos. ^‡^ Day 1 > Day 10. All values remained within the normal reference range for horses. ^§^ Day 1 < Day 10. All values remained within the normal reference range for horses. ^¶^ Abbreviations of variables are as follows: ALP (alkaline phosphatase); ALT (alanine aminotransferase); AST (aspartate aminotransferase); GGT (gamma-glutamyl transferase); BUN (blood urea nitrogen); TCO_2_ (bicarbonate).

**Table 7:** Intake, fecal output, and digestibility of dry matter, crude protein, and neutral detergent fiber in *n* = 3 black rhino. Measurements did not differ between control and HBED treatments, so values were pooled across treatments (mean ± SEM).

	Units	DM ^†^	Crude Protein	NDF ^†^
Intake	g DM/kg BW ^†^	20.1 ± 0.8	2.7 ± 0.2	12.4 ± 0.1
Fecal Output	g DM/kg BW	13.4 ± 2.6	1.0 ± 0.1	9.3 ± 1.4
Digestibility	%	35.4 ± 4.6	61.5 ± 1.9	25.2 ± 4.2

^†^ Dry matter (DM); body weight (BW); neutral detergent fiber (NDF).

**Table 8:** Urine and fecal iron concentrations, iron intake, excretion, and digestibility in *n* = 3 black rhino after supplementation with HBED or Control for 10 days.

	Units	Control	HBED	SEM	*p*-Value
Iron Intake	mg/kg BW	2.6	2.4	0.3	0.66
Fecal Iron Excretion	mg/kg BW	7.6	8.2	5.6	0.94
Iron Digestibility	%	−188.2	−237.5	−112.0	0.84
Urine Iron	µg/dL	73.5	382.5	21.9	0.006
Fecal Iron	mg/kg DM	555.4	502.8	100.4	0.75

**Table 9:** Measures of iron status in *n* = 3 black rhino after supplementation with HBED or Control for 10 days.

			Treatment			*p*-Values		
	Units	Day	Control	HBED	SEM	Trt	Time	Time × Trt
Serum non-heme iron	µg/dL	0	228.6	213.6	22.9	0.81	0.19	0.87
		10	267.6	261.7				
Total iron binding capacity	µg/dL	0	379.2	374.5	10.3	0.52	0.01	0.21
		10	413.6	447.0				
Transferrin saturation	%	0	61.1	57.7	5.4	0.63	0.85	0.79
		10	64.3	57.1				
Ferritin—males only	ng/mL	0	403.0	346.5	20.7	0.42	--	--
		10	395.5	421.5				
Ferritin—female only	ng/mL	0	--	1615	--	--	--	--
		10	1243	14,158				

## Data Availability

The original contributions presented in this study are included in the article. Further inquiries can be directed to the corresponding author.
